# The unique evolutionary pattern of the Hydroxyproline-rich glycoproteins superfamily in Chinese white pear (*Pyrus bretschneideri*)

**DOI:** 10.1186/s12870-018-1252-2

**Published:** 2018-02-17

**Authors:** Huijun Jiao, Xing Liu, Shuguang Sun, Peng Wang, Xin Qiao, Jiaming Li, Chao Tang, Juyou Wu, Shaoling Zhang, Shutian Tao

**Affiliations:** Centre of Pear Engineering Technology Research, State Key Laboratory of Crop Genetics and Germplasm Enhancement, Nanjing Agricultural University, Nanjing, 210095 China

**Keywords:** Evolutionary pattern, Expression divergence, Gene duplication, HRGPs, Hyp-rich motif, Positive selection

## Abstract

**Background:**

The hydroxyproline-rich glycoprotein (HRGP) superfamily, comprising three families (arabinogalactan-proteins, AGPs; extensins, EXTs; proline-rich proteins, PRPs), is a class of proline-rich proteins that exhibit high diversity and are involved in many aspects of plant biology.

**Results:**

In this study, 838 *HRGPs* were identified from Chinese white pear (*Pyrus bretschneideri*) by searching for biased amino acid composition and conserved motifs. 405 *HRGPs* were derived from whole genome duplication (WGD) events which is suggested to be the major force of driving *HRGPs* expansion and the recent WGD event shared by apple and pear generated most duplicated *HRGPs* in pear. This duplication event drived the structural variation of the *HRGPs* encoding hydroxyproline (Hyp)-rich motifs. The rate of HRGPs evolution mainly impacted the Hyp-rich motifs even in chimeric HRGPs. During the evolution of 53 *PRPs* that are also typified by 7-deoxyloganetin glucosyltransferase-like genes, the duplication from *PRP* to *non-PRP* was indirectly modified by positive selection. These results suggested that the rate of *HRGP* evolution mainly influenced the Hyp-rich motifs even in chimeric *HRGPs*. The expression divergence of *HRGPs* was higher than that of other commonly duplicated genes. In pear pistil, 601 *HRGPs* exhibited expression, while in pear pollen, 285 *HRGPs* were expressed. The qPCR results revealed that *Pbr036330.1* and *Pbr010506.1* showed different expression profile in self-incompatibility of pear pistil.

**Conclusions:**

The researches indicated that WGD events was the main duplication type during the evolution of HRGPs, and the highly variable Hyp-motifs might be accountable for the expansion, evolution and expression divergence of *HRGPs* and that this divergence may be responsible for the gain of new functions in plants.

**Electronic supplementary material:**

The online version of this article (10.1186/s12870-018-1252-2) contains supplementary material, which is available to authorized users.

## Background

Hydroxyproline-rich glycoproteins (HRGPs) is a superfamily that contains three subfamilies: highly glycosylated arabinogalactan-proteins (AGPs), moderately glycosylated extensins (EXTs) and lightly glycosylated proline-rich proteins (PRPs) [1, 2], all of which are usually post-translationally modified by prolyl 4-hydroxylase, generating hydroxyproline (Hyp) residues. HRGPs are expressed widely in the plant kingdom, from algae to flowering plants, indicating that they play fundamental roles in plant lifes [3]. Indeed, HRGPs are thought to exhibit broad functionality, from providing structural integrity and mediating cell-cell interactions to facilitating intercellular communication in roots, leaves and reproductive tissues. Pear fertility is a key concern due to the fact that pear is a major commercial crop, and the role of HRGPs in the reproductive development of the pear is of particular interest.

The arabinogalactan chains of AGPs are usually linked to galactose by Hydroxyproline-Galactose (Hyp-Gal) linkages. Recent studies demonstrated that AGPs play vital role in many tissues, such as leaves, roots and floral tissues [4]. AGPs often lie in the cell surface and are implicated in vegetative and reproduction growth [2, 5]. T. Schindler reported that AGPs are involved in plant development, such as the xylem differentiation [6]. AGP31 (At1g28290) participates in cell wall formation by interacting with various cell wall components [7]. AGP19 (At1g68725) is involved in cell division and expansion [8]. AGPs expressed in the transmitting tissue of the style also assist pollen adhesion on the stigma and direct pollen tube growth along the transmitting tissue [9, 10]. AGPNa3 protein (RT35 protein) is localized in the stigma, specifically in the pistil of *Nicotiana alata* (tobacco) [11]. 120 kDa glycoprotein (120 K) expressed in the style plays a role in S-specific pollen rejection in *N. alata* and is taken into the pollen tube [12]. The AGP-enriched, stylar-transmitting canal exudate of *Lilium longittorum* has been shown to serve as an adhesive matrix that enhances pollen tube growth in vitro [13].

EXTs contains several repetitive SP_n_ motifs in their Hyp-rich motifs. Most EXTs belonging to insoluble cell wall proteins are expressed in root, pollen/stamen or siliques [14]. EXTs are involved in nearly all aspects of plant growth and development, including pollen recognition, fertilization [15], defence responses [16] and cell division, differentiation and elongation [17–19]. The repetitive SP_n_ motifs of Pex protein may participate in species-, family- or tissue-specific recognition in *Solanum lycopersicum* (tomato) [20]. Pex1 expressed in *Zea mays* pollen interacts with a specific partner and triggers the downstream reaction [21]. AtLRX1 is directly involved in the process of cell wall formation in *Arabidopsis thaliana*. The SP_n_ motifs in LRX proteins with variable length and number are involved in regulating signalling processes in cell walls [22].

Proteins rich in prolines or Hyps cannot be classified into EXTs or AGPs, and they are defined as PRPs [23]. PRPs are mostly expressed in endosperm, shoot apex and petiole, while a few are expressed in root [14] or are involved in reproduction. PRPs in *Glycine max* (SbPRP1, SbPRP2, SbPRP3) are expressed in various kinds of cells and are distinct from cell wall proteins. ENOD2 isolated from *Pisum sativum* is important for cell morphology, especially in the nodule parenchyma [24]. HyPRP in maize is highly expressed in zygotic embryo and ovary prior to pollination and is thought to contribute to the stability and defence of the developing embryo [25].

Gametophytic self-incompatibility (SI) is a widespread mechanism that prevents inbreeding and promotes out-crossing in flowering plants [26]. SI of plants belonging to the genera *Rosaceae*, *Solanaceae* and *Plantaginaceae* is determined by S-RNase [27]. Pollen tube growth in the extracellular matrix (ECM) triggers a series of interactions between pollen and pistil. For instance, AGP components of the stylar ECM directly interact with pollen tubes in tobacco [12]. AGPNa3 protein (RT35 protein) located in the stigma may have a specific role in pistil [11]. Transmitting tissue-specific glycoprotein (NaTTS) was reported to be involved in attracting pollen tubes in vitro and stimulating pollen tubes both in vivo and in vitro. NaTTS protein levels are highly reduced in transgenic tobacco plants, which leads to a reduction of pollen tube growth rate [28, 29]. The class III pistil-specific extensin-like (PELPIII) proteins were reported to incorporate into both compatible and incompatible pollen tube [12, 30]. 120 K is taken into pollen tube and is associated with pollen tube vacuoles [29, 31]. 120 K, PELPIII and TTS are able to bind S-RNase, while only 120 K is necessary for SI [12, 28, 29]. However, the role of most HRGPs in the SI of pear remains unclear.

Herein, we studied pear HRGPs by investigating phylogenetic relationships, evolutionary patterns and expression patterns in reproductive tissues. In contrast to the bioinformatics analysis of HRGPs in *Arabidopsis* and *Populus trichocarpa* (black cottonwood) [32, 33], our data suggested the evolution patterns and expression divergence influenced by Hyp-rich motifs and elucidated the importance of Hyp-rich motifs in *HRGPs* evolution. Finally, the expression patterns of *HRGPs* in reproductive tissues were analysed to uncover their functional roles in SI.

## Results

### Identification and classification of HRGPs in pear

In order to collect all *HRGPs* in pear, multiple search methods based on the features of each family were utilized. Based on biased amino acid composition and conserved motifs, a total of 838 *HRGPs* were identified, including 522 *AGPs*, 201 *PRPs* and 115 *EXTs* (Additional file 1: Table S1). However, 56 *HRGPs* were identified as repetitive sequences during screening. Based on the characteristics of Hyp-rich motifs, HRGPs were classified into three families: AGPs, EXTs and PRPs.

The percentage, sort order and the amount of proline (P), alanine (A), serine (S) and threonine (T) were regarded as criteria for identifying AGPs, and 522 sequences were collected (Table 1). By a detailed identification of sequence characters, the AGP family was divided into five subfamilies: AG (arabinogalactan) peptides, lysine-rich AGPs, FLAs (fasciclin-like AGPs), EXT-AGPs and classical AGPs. 126 AG peptide sequences were identified by searching for biased amino acid (aa) compositions with at least 10% PAST and protein length less than or equal to 90 aa. 183 Lys-Rich AGPs were identified by searching for biased aa compositions with at least 10% PAST and lysine-rich motifs. 7 FLAs proteins containing fasciclin H1 motifs with more than 10% PAST were identified. 54 EXT-AGPs were identified by searching for [SP]_3,4,5_ motifs with at least 10% PAST content. 152 Classical AGPs were identified by searching for biased aa compositions with at least 30% PAST and no swapped domains. Comparing the size of the subfamily, members of Lys-Rich AGPs > Classical AGPs > AG peptides > EXT-AGPs, and only 1% of AGPs were FLAs. The signal peptides (SPs) cleavage sites and glycosylphosphatidylinositol (GPI) anchor sites were also predicted. 25% and 9% of AGPs members were annotated with SPs and GPI anchors, respectively. The prototypical structure of each subfamily member is displayed in Additional file 2: Fig. S1.

**Table 1:** Identification and classification of HRGPs in pear genome

Family	Subfamily	Searching Strategies	HRGPs with SP	HRGPs with GPI	Total
EXT	SP3	> = 2 SPPP	19	3	56
	SP4	> = 2 SPPPP	16	2	33
	SP5	> = 2 SPPPPP	16	0	26
AGP	EXT-AGPs	> 10%PAST& SP motif	33	4	54
	AG-peptides	> 10% PAST & AA < 90	15	5	126
	lysine-AGPs	> 10%PAST& Lysine rich Domain	33	10	183
	FLAs	> 10%PAST&Fasciclin Domain	5	3	7
	Classical AGPs	> 30% PAST	44	24	152
PRP	PRP	PPV.[KC]	17	0	201

Note: SP refers to signal peptide; GPI refers to glycosylphosphatidylinositol

115 EXTs genes were identified by screening pear protein database with more than 2 SP_3_, SP_4_ and/or SP_5_ motifs. EXT family members were divided into three subfamilies based on the number of Hyp-rich repeats in the motifs of SP_3_-EXT, SP_4_-EXT and SP_5_-EXT. The SP_3_-EXT subfamily consisted of 56 members, of which 19 have SPs cleavage sites and 3 have GPI anchor sites. The SP_4_-EXT subfamily consisted of 33 members and 16 have SPs cleavage sites and 2 have GPI anchor sites among them. The SP_5_-EXT subfamily consisted of 26 members and 16 of them have SPs cleavage sites and none has a GPI anchor site (Table 1). The typical structure of the EXT family is displayed in Additional file 2: Fig. S1.

201 PRPs were identified by screening [KKPCPP] and [PPVX(K/T)] motifs against the pear protein database. Only 17 PRPs members were predicted to have SPs, while none was found to have a GPI anchor site (Table 1). The typical structure of PRP family is displayed in Additional file 2: Fig. S1. The classification results suggested that our searching strategy is accurate.

### Phylogenetic analysis of the HRGP superfamily

**Fig. 1:**
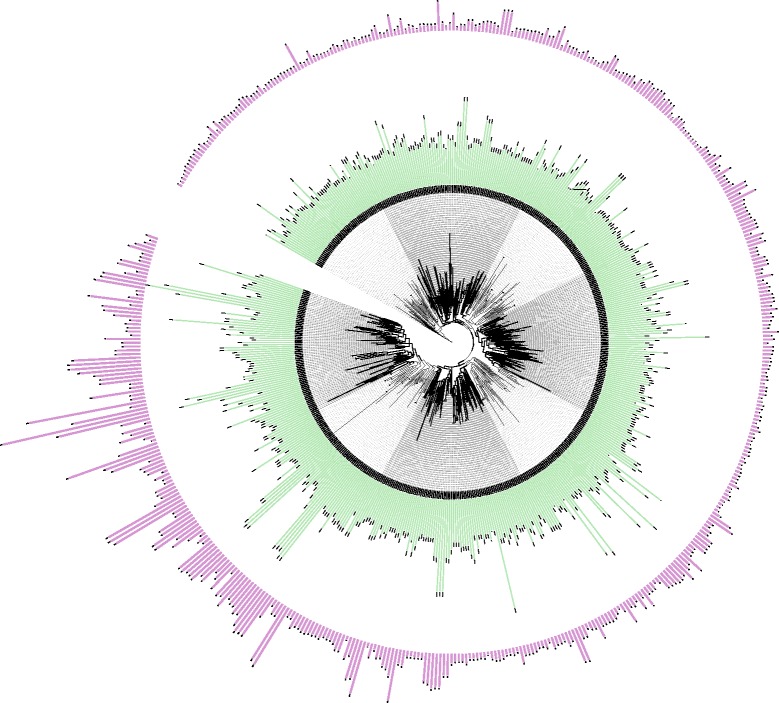
Phylogenetic tree of the AGP family. Green columns indicate the percentage of P, A, S and T in each AGP; pink columns indicate the total number of P, A, S and T in each AGP

Considering that the sequences structure of HRGPs from different families vary greatly, the phylogenetic tree of total superfamily members can not reflect the influence of biased aa composition on phylogenetic relationships. To survey phylogenetic relationships, the phylogenetic trees of each family were constructed using RAxML. The phylogenetic tree of AGP family was constructed based on the percentage of PAST and absolute number of PAST sequences in each member. Members of each subfamily did not cluster together, suggesting that the phylogenetic relationship was not affected by biased amino acid composition. However, AGPs with similar amount of PAST always clustered together, while AGPs with contrasting percentages of PAST showed a balanced clustering (Fig. 1). The phylogenetic tree of EXT family was constructed and combined with numbers of SP_n_ motifs (Additional file 3: Fig. S2). Members of each EXT subfamily spread evenly between the clades, suggesting that the phylogenetic relationship was not in accordance with the type of SP_n_ motifs. However, EXTs with relatively more SP_n_ motifs tended to cluster together.

The phylogenetic tree of PRP family was also constructed using amino acid sequences (Additional file 4: Fig. S3). Most PRPs with PPV[X]C motifs clustered together and were mainly distributed in two branches. PRPs with PPV[X]K clustered in a very scattered manner, suggesting that PRPs with this Hyp-rich motif may have more extensive functionality. The topological structures of three phylogenetic trees suggested that the evolutionary pattern of HRGPs is complexity.

### Expansion patterns of the HRGP superfamily

**Fig. 2:**
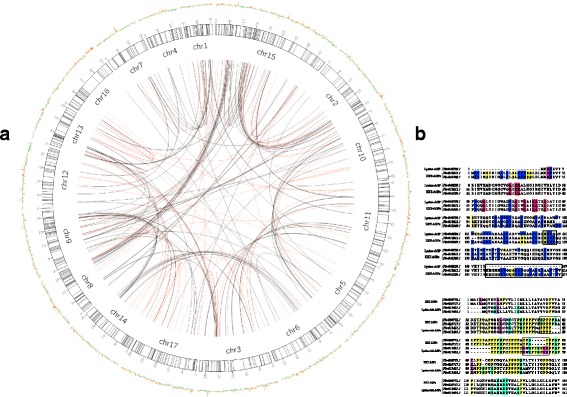
Gene duplication analysis of *HRGPs* in pear. **a**. Gene pairs within *HRGPs* are joined by red lines; gene pairs in which only one duplicated gene is an *HRGP* are joined by black lines. Average GC content is indicated by coloured columns outside the chromosomes. Regions with high GC content are indicated by red columns, and low GC regions are indicated by green columns. **b**. The alignments of two collinear gene pairs between *EXT-AGPs* and *lysine-rich AGPs*. The mutant amino acid sequences are indicated by black boxes

**Fig. 3:**
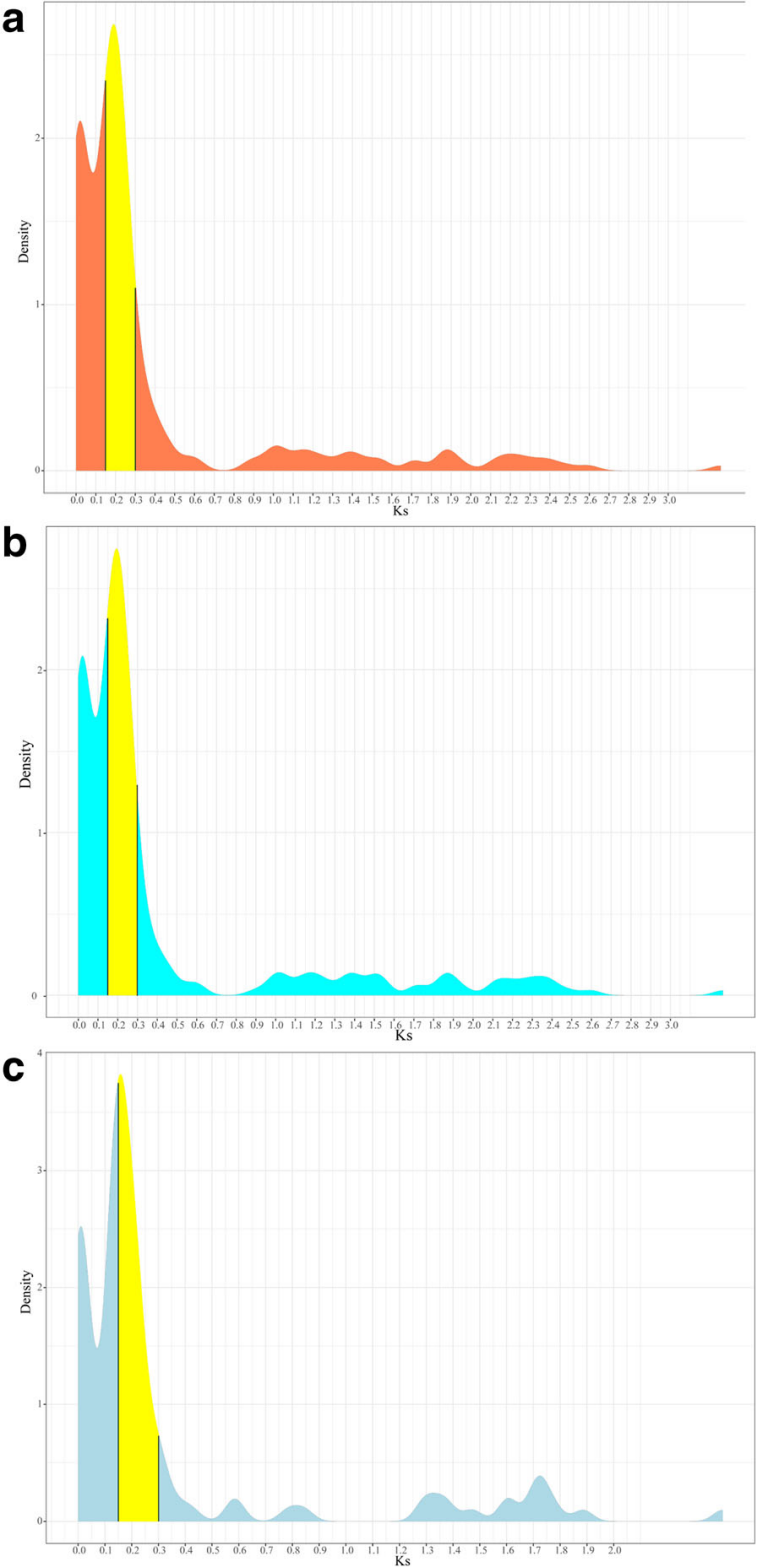
The relative duplication time of *HRGPs* as indicated by Ks values. **a**-**c**. The relative duplication time of the *AGP* (A)*, EXT* (B) and *PRP* (C) families. The yellow region ranging from 0.15 to 0.3 indicates the recent WGD events shared by pear and apple

To further ascertain the underlying discordance mechanism between phylogenetic relationship and biased aa composition, we determined the expansion patterns of *HRGPs*. It has been demonstrated that whole genome duplication (WGD), segmental duplication and single-gene duplication are the major gene duplication modes [34]. Five modes of gene duplication in the HRGP superfamily were identified using MCSanX, including WGD, segmental, tandem, dispersed and proximal duplication. The duplication types for HRGP family members were diverse (Table 2). Most duplicate gene pairs among *HRGPs* were derived from WGD event, but a large proportion of single-copy *HRGPs* and dispersed duplication *HRGPs* were also detected, and suggested that WGD and dispersed duplication mostly accounted for the expansion of *HRGPs*. The duplicate gene retention and evolution after WGD were heavily influenced by gene features such as structural complexity or GC content [35]. The genomic GC content was calculated in a 10 kb window and displayed beside the chromosomes by Circos (Fig. 2a). Genes with GC-rich regions typically exhibited a faster rate of evolution [36]. Indeed, we found that nearly half of the *HRGPs* located in the GC-rich regions evolved rapidly. Ninety-three collinear gene pairs were found among all *HRGPs*, while no PRP gene was found to be collinear with other *HRGPs*. Collinear gene pairs were located in homologous chromosome regions, such as 9 and 17, 8 and 15, which descended from the recent WGD event occurred in pear [37]. This result indicated that the recent WGD event shared by pear and apple generates numerous *HRGPs* (Fig. 3). We observed a number of gene pairs in which the two gene copies belonged to different subfamilies of *HRGPs,* indicating frequent structure variations within the family. According to the sequence alignments of chimeric HRGPs, we found that Hyp-rich motifs were much more variable than common conserved domains and might be responsible for the high variation of HRGPs after gene duplication.

**Table 2:** Statistics of duplication types of *HRGPs* in pear

Family	Subfamily	Duplication type				
		Dispersed	Proximal	Singleton	Tandem	WGD
AGPs	AG peptides	33	10	54	9	20
	Classical AGPs	28	4	36	7	77
	FLAs	2	2	0	0	3
	EXT-AGPs	15	0	9	2	28
	Lysine AGPs	30	12	24	12	105
EXTs	SP3-EXT	18	3	1	2	32
	SP4-EXT	7	0	2	2	22
	SP5-EXT	8	1	7	0	10
PRPs	–	48	26	9	10	108
Total	838	189	58	142	44	405

We then performed sequence alignment of collinear gene pairs in which the two gene copies belonged to different subfamilies in order to analyze the structural variation of HRGPs such as Pbr028717.1 (EXT-AGP), Pbr013421.1 (lysine-rich AGP), Pbr030772.1 (EXT-AGP) and Pbr013433.1 (lysine-rich AGP). The results demonstrated that P_52_ in the two lysine-rich AGPs had mutated to S_56_, putting Pbr030772.1 and Pbr028717.1 into the EXT-AGPs subfamily. In addition, the loss of K_67_, K_74_ and K_75_ resulted in the absence of lysine-rich motifs in Pbr030772.1 and Pbr028717.1 (Fig. 2b). Additionally, *Pbr042269.1* (*EXT-AGP*) were collinear with *Pbr012012.1* (*EXT-AGP*) and *Pbr040388.1* (*lysine-rich AGP*). Each of them contained a typical lysine motif of KIVKAIKKTRK, while both Pbr042269.1 and Pbr012012.1 contained two extra SPPP motifs. Compared with the two EXT-AGPs, P_129_ in Pbr040338.1 mutated into H_129_ and a set of amino acids marked with a black box were deleted, including an SPPP motif. These mutations changed *Pbr040338.1* into a typical lysine-rich AGP (Fig. 2b). These sequence variations between duplicate pairs among HRGPs indicated that the Hyp-rich motif was particularly variable and affected AGP classification. Furthermore, the duplication between HRGPs and non-HRGPs was much more frequent than one HRGP to another HRGP, which also confirmed the high frequency of variance in Hyp-rich motifs (Fig. 2a).

### Evolutionary patterns of HRGPs

Many HRGPs are chimeric proteins formed via domain swapping [38]. Considering that most conserved domains evolve under purifying selection while the variation of Hyp-rich motifs in HRGP is frequent, we explored whether Hyp-rich motifs would affect the evolutionary rate. Comparisons of evolutionary rates between chimeric *HRGPs* and genes with corresponding domains elucidated special duplication patterns by collinearity analysis.

**Fig. 4:**
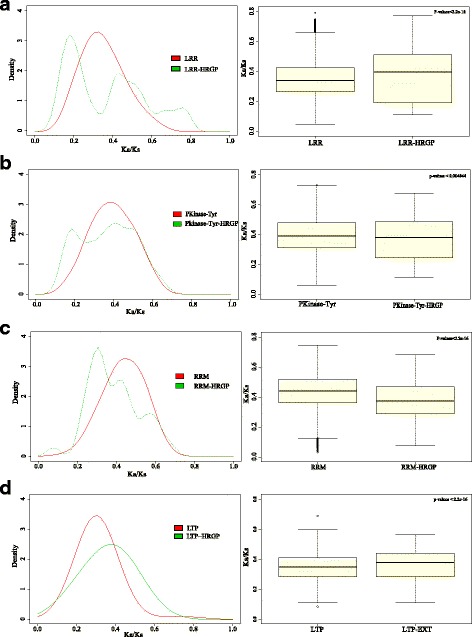
Comparison of evolutionary rates between chimeric *EXTs* and corresponding homology genes without proline-rich motifs. **a** Comparison of evolutionary rates between LRR family and LRR-HRGP family. **b** Comparison of evolutionary rates between Pkinase-Tyr family and Pkinase-Tyr-HRGP family. **c** Comparison of evolutionary rates between RRM family and RRM-HRGP family. **d** Comparison of evolutionary rates between LTP family and LTP-HRGP family. Abbreviations: LRR: leucine-rich domain; Pkinase-Tyr: protein tyrosine kinase; RRM: RNA recognition motif; LTP: liquid transfer domain

The ratio of Ka (nonsynonymous substitutions per site) and Ks (synonymous substitutions per site) was widely used to estimate the evolutionary rate of coding sequences. We chose a set of chimeric HRGPs containing conserved domains for further analysis, such as leucine-rich repeats (LRR, PF12799.1), Pkinase_Tyr (PF07714.15), probable lipid transfer (LTP, PF14368.4) and RNA recognition motifs (RRM, PF00076.20). The selected HRGPs were used to compare the evolutionary rates between chimeric HRGPs and proteins with similar swapped domains (Fig. 4). We found that the evolutionary rates between chimeric HRGPs and genes with corresponding domains were significantly different. Specifically, for chimeric HRGPs with LRR and LTP motifs, the evolutionary rate of HRGPs was higher than that of non*-*HRGPs. For chimeric HRGPs with PKinase_Tyr and RRM, the evolutionary rate of HRGPs was lower. It suggested that in different chimeric HRGPs, the Hyp-rich motif did not simply accelerate or decelerate the evolutionary rate. We also compared the evolutionary rate among chimeric HRGPs, and no significant difference was found by T-test. It suggested that Hyp-rich motifs were likely to be more influential than conserved domains in chimeric HRGPs on the evolutionary rate.

Three phylogenetic trees were constructed via RAxML using aa sequences. Genes clustered in each branch presented a close phylogenetic relationship. Analyzing the PRP phylogenetic tree and the Hyp-rich motif features of each branch, we found that most PRPs with a PPV[X]C motif belonged to a specific gene family (Additional file 4: Fig. S3).

To explore the formation of chimeric HRGPs, we analyzed the 7-deoxyloganetin glucosyltransferase-like gene family containing both PRPs and non-PRPs as a representative set. Using PRP Pbr024572.1 as the query sequence to blast against the pear proteome, we found 266 homologous genes belonging to 7-deoxyloganetin glucosyltransferase-like gene family. Fifty-three closely related genes containing both PRPs and non-PRPs were retained. We found PRPs spread among all phylogenetic branches, indicating that an ancient gene may have contained this motif.

**Fig. 5:**
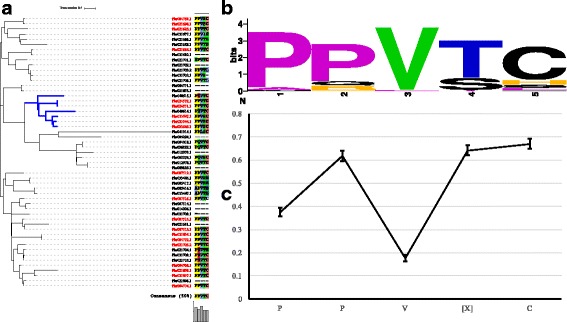
The variation of Hyp-rich motifs in PRPs is affected by selective pressure. **a**. The phylogenetic tree and corresponding motifs of PRPs and non-PRPs. Red taxa refer to PRPs and black taxa refer to non-PRPs. **b**. Amino acid compositions of 53 PRPs and non-PRPs. **c**. Comparison of omega among all sites within the motif

**Fig. 6:**
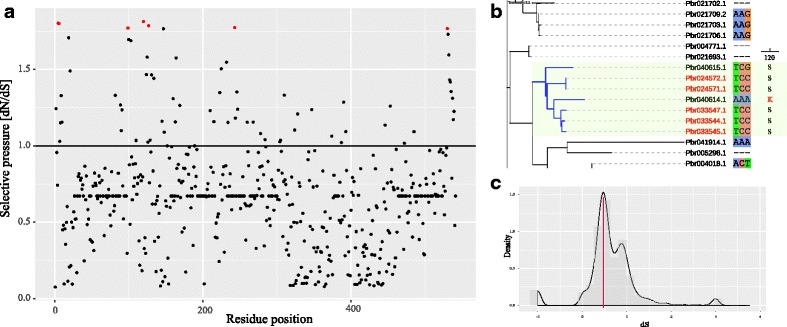
The evolution pattern of *PRPs* in the *7-deoxyloganetin glucosyltransferase* family. **a**. The selective pressure acting on total amino acid sites. The significant positively selected sites are marked by red circles. **b**. The positively selected site S_120_ mutated to K_120_ among PRPs and non-PRPs. Red taxa refer to PRPs and black taxa refer to non-PRPs. **c**. The divergence time of 7-deoxyloganetin glucosyltransferase family. The red line refers to 0.4871, indicating the divergence time of PRPs and non-PRPs in the blue clade

We used codeml from the PAML package to estimate the selection pressure for 53 genes (Fig. 5a). We found that most of the sites were under negative selection (Fig. 6a), suggesting that genes in the tree evolved conservatively; however, 7 amino acid sites under positive selection were also found. In the blue clade, most of the genes were identified as PRPs, while Pbr040614.1 mutated into a non-PRP. Among all estimated sites, one positively selected amino acid site S_120_ in Pbr040614.1 mutated to K_120_ (Fig. 6b). The mutation of this site may trigger a change of Pbr040614.1 in function and result in the structural variation of its Hyp-rich motif. However, the PRP Hyp-rich motif PPV[X]C was under significant purifying selection, implying that the motif of PPV[X]C was conservative (Fig. 5b, c). It seemed that this Hyp-rich motif was not directly modulated by positive selective pressure, while other sites under positive selection could lead to the structural variation of Hyp-rich motif in PRPs.

The divergence time of the blue clade ranged from 0.45 to 0.52, during which 7-deoxyloganetin glucosyltransferase-like genes of pear experienced an explosive expansion (Fig. 6c). The divergence time of *Pbr040614.1* and the other *PRPs* ranged from 0.46 to 0.49 and was also within the time of rapid expansion. We also found that during the expansion mode of 7-deoxyloganetin glucosyltransferase-like genes, there was no WGD event which is a major force to expand most gene families. The explosive expansion might have been caused by numerous single-gene duplications, and nearly half of the identified *PRPs* were found to duplicate in this manner.

**Fig. 7:**
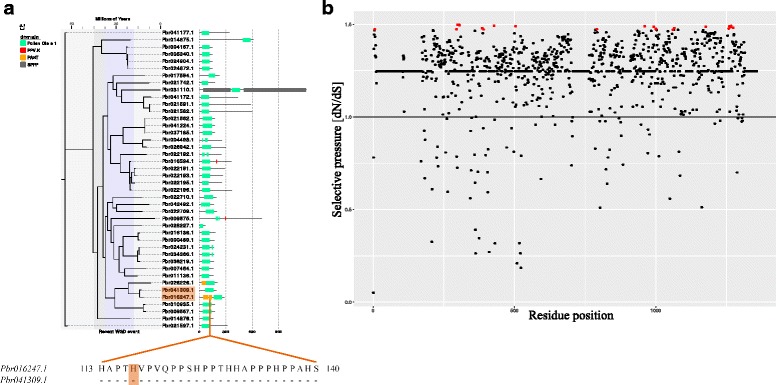
Selective pressure promotes the structure variation of chimeric *HRGPs*. **a**. The selective pressure acting on total amino acid sites. The statistically significant positively selected sites are marked by red circles. **b**. A phylogenetic tree constructed from 42 sequences containing the Pollen Ole e I domain among all chimeric HRGPs. Violet refers to the recent WGD event and Orange H indicates the positively selected amino acid site

*HRGPs* from Pollen Ole e I family typically participated in the highly divergent reproductive process in plants, making the duplication of this chimeric HRGP domain particularly important. The Pollen Ole e I domain HMM file was used as a query to perform the Hmmsearch against the pear genome. We found 42 sequences containing two PRPs, two AGPs and one EXT. The phylogenetic tree of the 42 genes was constructed and the selection pressure on aa residues was estimated. It was found that the Pollen Ole e I gene family was under positive selection and 23 amino acid sites were under strong selective pressure (Fig. 7b). We also found that *Pbr026226.1*, *Pbr041309.1* and *Pbr016247.1* were closely related in the clade, but the *Pbr041309.1* was not an *AGP*. Sequence alignment suggested that Pbr041309.1 lacked an N-terminal PAST sequence. We also found that a positively selected site, H_117,_ was lost in Pbr041309.1, while the other two genes retained this site (Fig. 7a). We speculated that the mutation of H_117_ might have triggered the loss of PAST fragment. These results suggested that positive selection is correlated with the gain and loss of *HRGPs*.

### Expression of HRGP superfamily genes in reproductive tissues of pear

Because the evolutionary rates between chimeric *HRGPs* and genes with corresponding domains showed divergent patterns, we analysed expression pattern to further explore special chimeric HRGPs in pear. Many HRGPs were reported to play vital roles in reproductive processes, particularly in styles [39]. Therefore, we examined the expression patterns of *HRGPs* in pollens and styles of pear, which is a classic SI plant. To better understand the expression patterns of *HRGPs*, we used the RPKM values from RNA-seq in different developmental stages of the pollen tube and during the dynamic development of self- and non-self-pollinated styles (The data have not been published).

A diverse expression pattern of *HRGPs* in different tissues was found. We found 601 *HRGPs* were expressed in pollinated pistils, while only 285 were expressed in pollen. We clustered all expressed *HRGPs* from both transcriptome libraries (Additional file 5: Fig. S4) and found that *HRGPs* represented distinct expression patterns. In pollen, the highly expressed HRGP genes were less than low expressed genes, while it was opposite in pollinated style (Additional file 5: Fig. S4). This suggested that HRGP genes may play more important roles in pistils than that in pollens. These results were in accordance with previous data of TTS and 120 K [40]. We also found that the expression of most *HRGPs* was stable during different stages of pollen tubes and pistils development. We also carried out the analysis of expression pattern of *HRGPs* in fruit development and we found that the *HRGPs* showed complicated expression patterns (Additional file 6: Fig. S5).

‘Jinzhui’ is a self-compatible bad mutant from ‘Yali’ and we hypothesised that the mutant genetic features might be related to the pollen. Previous report indicated that pollination in ‘Jinzhui’ × ‘Yali’ failed, while pollination in ‘Jinzhui’ × ‘Jinzhui’ was successful [41]. However, other reports suggested that ‘Jinzhui’ pollen could pollinate the pistils of both ‘Jinzhui’ and ‘Yali’ [42]. Therefore, we analyzed RNA-seq of various stages of the two different pollinated pistils to reveal potential mutation mechanism between ‘Yali’ and ‘Jinzhui’. It may be caused by the divergent expression pattern of HRGP genes during the pollination process.

To test this possibility, the pistils of ‘Jinzhui’ were pollinated with ‘Jinzhui’ or ‘Yali’ pollen separately and were collected at 24 h, 48 h and 72 h after pollination. After comparing the reads per kilobase per million (RPKM) of the HRGP superfamily, two HRGP genes exhibited differential expression patterns.

In ‘Jinzhui x Jinzhui’ pistils after 24 h post-pollination, the expression level of *Pbr036330.1* was up regulated and more than that in ‘Jinzhui x Yali’, while *Pbr010506.1* expression was down regulated. However, the differential expression of the two *HRGPs* reduced in pistils after 48 h post-pollination and there was no difference at 72 h post-pollination. The qRT-PCR results were consistent with the RPKMs tendency in transcriptome data of pollinated pistils (Additional file 7: Fig. S6A, B). Considering that at 24 h post-pollination, the self-compatible and incompatible reactions in pear pistils had already occurred [43], and that these reactions would cease at 48 h. The data suggested that this differential expression pattern may associate with mutations of mechanisms downstream of the pollination self-compatibility reactions.

**Fig. 8:**
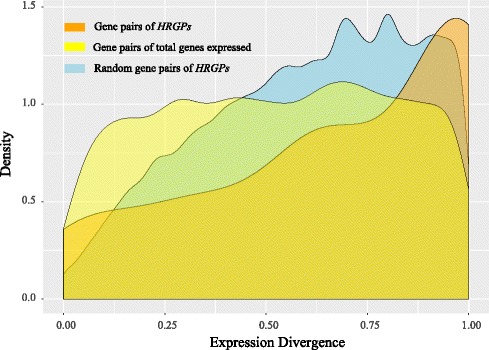
Comparison of the expression divergence among HRGP gene pairs, randomly paired HRGP genes and total gene pairs. The gene pairs of totally expressed genes were acquired by MCScanX. The expression profile employed in this analysis was the RPKM value of pollinated pistils

Duplicated genes within a gene pair exhibiting high homology tended to display a similar expression pattern, while our results indicated that relatively lower homology was found between HRGP gene pairs following the expansion of the superfamily. To better elucidate the characteristics of *HRGP* expression divergence, we examined random HRGP gene pairs versus total gene pairs (as controls). Expression divergence of HRGP genes randomly combined as gene pairs indicated the universal expression divergence among *HRGPs* and total gene pairs acquired from the Plant Genome Duplication Database (PGDD) indicated the universal expression divergence between duplicated genes. We found that random gene pairs, duplicated gene pairs and duplicated HRGP gene pairs presented distinct expression divergence features (Fig. 8). Expression divergence of total duplicated gene pairs likely reflected common rate of expression divergence, while expression divergence of random HRGP gene pairs was represented when there was no collinear relationship. Our results indicated that the expression of *HRGPs* without collinear relationships was more divergent than genes from duplicated pairs. However, expression analysis of HRGP gene pairs exhibited higher divergence than both random HRGP gene pairs and total expressed gene pairs. It suggested that duplication events of *HRGPs* may enhance their expression divergence.

To investigate the mechanism of expression divergence of *HRGPs*, we also analyzed the promoter sequences of HRGP genes from WGD gene pairs. For *HRGPs* from WGD showed a high level of expression divergence, we compared the promoter sequences of HRGPs gene pairs from WGD. we extracted the upstream 1000 bp of HRGPs genes and predicted transcription factor bonding sites/cis elements present in the 1000 bp upstream region. The results suggested that HRGPs gene pairs with higher expression correlation shared a couple of transcription factors while HRGPs gene pairs with higher expression divergence had no common transcription factor (Additional file 1: Table S2). It is inferred that the variation of promoter sequences in *HRGPs* may be an important factor accounting for expression divergence of HRGPs gene pairs from WGD events.

## Discussion

### A more sensitive approach of identifying HRGPs

Our examination of the expansion, evolution and expression of the HRGP gene superfamily in pear reproductive tissues highlighted key differences from those of other gene families. We employed a biased aa composition analysis for sequence collection of the HRGP superfamily instead of Blastp and Hidden Markov Model (HMM) typically used for other gene families. We employed a family-specific strategy for each family within the HRGP superfamily. The Hyp-rich motifs of each HRGP were also annotated (data not shown). Through this process, 838 genes were identified, far more than the number identified by other previously reported strategies. Indeed, in *P. trichocarpa,* 271 *HRGPs* were identified, while in *Arabidopsis* 162 were identified [14, 33]. To compare our searching method with that previously reported, we searched the *HRGPs* in Arabidopsis and blackcotton wood and more *HRGPs* were acquired with our method (Additional file 1: Table S3). Although our search strategies were based on biased aa composition searching patterns of Showalter [14], a perl script written by us were applied to screen HRGPs and the searching strategy was modified to maximise the identification of HRGPs in pear. During the screening process, we used different percentage of PAST residues in candidate HRGPs using BlastP and found that a minimum of 10% was critical to identify HRGPs. Compared with BIO OHIO, our search method is more adaptive for identifying HRGPs.

### Both WGD and single-gene duplication are important for *HRGPs*

WGD events were prevalent in several angiosperm genomes [44]. Ancient WGD events took place in the ancestral genome of angiosperm plants [45], and some plants experiencing multiple lineage-specific WGD events [46, 47]. It suggested that WGD events was the major force of driving the expansion of gene families, and a large number of duplicate genes were retained from WGDs and segmental duplications [48]. However, single-gene duplication also occurred frequently in angiosperm plants [49]. Previous studies reported that pear and apple experienced two rounds of WGD events approximately in 140 MYA [50] and 30–45 MYA [51], with the two species diverging from each other in 5.4–21.5 MYA [37]. Our results indicated that these WGD events mainly contributed to the expansion of the HRGP family, and the extensive dispersed duplication found in HRGPs was also important. In addition, single-gene duplication events also accounted for a large proportion. An alternative explanation for the substantial proportion of dispersed duplicates detected in the HRGP family may be that duplicate genes derived from recent WGD events experienced chromosome rearrangement followed by single-gene duplication, resulting in a burst of dispersed gene duplication [52, 53]. It was reported that tandem duplication largely contributed to the expansion of some large gene families [54]; however, our results indicated that tandem duplication did not play a large role during the expansion of *HRGPs.*

### HRGPs are important for reproductive processes

Because HRGPs were reported in many aspects of flowering plant reproductive processes [12, 39], we explored their expression pattern in pollen development and different pollinated pistils. Previous studies showed that TTS protein, 120 K and PELPIII are stylar glycoprotein HRGPs that could bind S-RNase in vitro in tobacco and also could interact with pollen tubes [12]. Two EXTs, *Pbr036330.1* and *Pbr010506.1* were abundantly expressed in pollinated pistils. Furthermore, both HRGPs exhibited differential response to SI. In pollens, *Pbr036330.1* was expressed in low levels, while *Pbr010506.1* was not expressed. However, the two genes showed high RPKM values in both self- and non-self-pollinated pistils. Meanwhile, we found that *Pbr036330.1* and *Pbr010506.1* exhibited higher expression in self-pollinated pistils than in non-self-pollinated pistils at 24 h post-pollination. However, these differences reduced at 48 h, and were absent at 72 h (Additional file 7: Fig. S6A, B). Previous research suggested that SI in pear usually occurred during 24 h post-pollination [55]. We speculated that the response process of these two HRGPs might be related to SI. Of note, the protein structures of both HRGPs contained a SPs and SP motifs, while no other conserved domains were found. However, TTS, 120 K and PEPLIII, which both contained Pollen Ole e I domains, contained only a SP_n_ motif. The relationship of the two HRGPs and SI requires further study.

The *Pbr031110.1* belonged to EXT subfamily containing a particularly unique sequence of Pollen Ole e I domain. The relative expression levels of *Pbr031110.1* in both pollen and style were lower than that in fruit, leaf, ovary and stem, suggesting that Pbr031110.1 was not involved in the reproductive process unlike most HRGPs (Additional file 7: Fig. S6C). With regard to the rest of the analyzed pear genes, the expression level of *Pbr031110.1* was the highest in fruit, followed by ovary. Interestingly, through a blastp search of Pbr031110.1 against several species, including *Prunus persica*, *Malus domestica*, *Fragaria vesca*, *Prunus mume*, *Vitis vinifera* and *Arabidopsis thalinana*, no genes were found with a homology coefficient greater than 40% (data not shown). Pbr031110.1 contained a conserved Pollen Ole e I domain and numerous repetitive SP_5_ motifs which did not exist in other species, even in the closely related apple. HRGPs were associated with the polymerisation of lignin and stone cells, of which there was a particularly high content of lignin in pear fruit that was unique among flowering plants [56]. We speculated that Pbr031110.1 could account for this phenomenon.

### The variable Hyp-rich motifs of HRGPs impact plant evolution

Our analysis suggested that WGD events promoted the expression divergence of *HRGPs*. Because duplicated genes typically exhibited more divergent expression patterns than single-copy genes [57], we compared the expression divergence of duplicated *HRGPs* with other common duplicated genes. This comparison revealed that the expression of duplicated *HRGP* is more divergent than common duplicated genes. Because the duplicated genes likely performed unique functions in different tissues and developmental stages [58], we speculated that the higher expression divergence of *HRGPs* may due to their highly variable Hyp-rich motifs. The structure integrity of Hyp-motifs could also impact the evolutionary rates of genes. The duplicated genes contained the same domains, and the ones with additional Hyp-rich motifs exhibited distinct evolutionary rates. Selection pressure analysis of the Pollen Ole e I and 7-deoxyloganetin glucosyltransferase-like gene family suggested that gain and loss of Hyp-rich motifs are more frequent under positive selection.

The expression divergence of two genes was generally considered to be driven by random effects during a neutral-evolution model [59, 60]. However, the extremely high expression divergence of *HRGPs* suggested that the evolution of *HRGPs* did not occur under this model. Furthermore, the high variability of Hyp-rich motifs correlated with similar variability in the expression of *HRGPs* particularly in duplicated *HRGPs*. Genes retained after duplication likely gained new functions in a genome, further driving expression divergence [61, 62]. Therefore, a large number of *HRGPs* with high expression divergence likely constituted a major force in plant evolution.

## Conclusions

Our data elucidated the expansion, evolution and expression patterns of *HRGP* superfamily in pear. Given the strong signal of positive selection and the drastic expression divergence between duplicated HRGP genes, we proposed that the HRGPs may play important roles in the evolution of novel functions and adaption of plants. The HRGP genes were highly rich in the pistil which might provide an important point of view for us to study reproductive process of pear.

## Methods

### HRGP sequences collection

All gene sequences of Chinese White pear (*Pyrus bretschneideri*) were downloaded from the pear genome project (http://peargenome.njau.edu.cn/) [37]. The biased aa composition was a basic criterion for collecting HRGPs [14]. Different subfamilies of HRGPs possessed various Hyp-rich motifs. The content and percentage of PAST were the criteria used to search for AGPs. Other subfamilies were identified based on PAST percentage as well as the presence of unique motifs. For FLAs, the H1 motif was used to perform a Hmmsearch [14]. For EXT-AGPs, the SP_n_ motif was used as an additional search criterion. For classical AGPs, a minimum of 30% PAST content was required. For AG peptides, the percentage of PAST was at least 10%, and the peptide length was less than 90 aa. For lysine-rich AGPs, [X]KK, KKK and K[X]K residue sequences were used as additional criteria [14]. For EXTs, more than 2 SP_n_ (*n* = 3,4 and 5) sequences were required. For PRPs, the PPV[X][KC] sequence was required. Blastp was used to check sequences with a threshold around 10% PAST. The SP annotations were performed by SignalP4.0 [63] and the GPI predictions by big-PI Predictor [64, 65].

### Gene duplication analysis of HRGP genes

The collinear relationships of duplicated *HRGPs* in pear were acquired by MCScanX [44]. Collinear gene pairs were presented by Circos [66], and the genomic location of *HRGPs* was superimposed into the corresponding chromosomes, while unanchored *HRGPs* were not included in the collinearity analysis. To estimate the GC content of pear genome, we split each chromosome into 10,00 kb per window and calculated the GC content of each 10 kb window by a Perl script.

### Estimation of selective pressure and evolutionary rate

To compare the evolutionary rate between chimeric HRGP genes and the genes with corresponding conserved domains, KaKs_Calculator2.0 [67] was selected to calculate the pairwise Ka/Ks values by the NG method [68]. We collected the LRRs (PF12799.5), RNA recognition motif (PF00076.20), LTP (PF14368.4), protein tyrosine kinase (PF07714.15) and pollen proteins Ole e I like (PF01190.15) by a Hmmsearch against the pear genome, with the corresponding HMM files downloaded from the pfam database (http://pfam.janelia.org/) [69]. The program Codeml in PAML was used to estimate the selection pressure [70]. Sequence alignments were performed by Muscle and displayed by JalView with default parameters [71, 72]. Hyp-motifs of PRPs were drawn by Weblogo (http://weblogo.berkeley.edu/logo.cgi) [73]. Guide trees were constructed by RAxML using CDS with GTRCATI mode [74]. In codeml, Site model was used to evaluate positively selected sites. Models M0, M7 and M8 were selected to evaluate the probability of omega. The likelihood ratio test (LRT) = chi2(2 dl) = chi(abs(2*(Lnl7-Lnl8))). The Ks values were used to estimate the divergence time of Pollen Ole e I gene family. Because the Ks values of 7-deoxyloganetin glucosyltransferase-like gene pairs were insufficient to estimate the divergence time of the entire family, Ks values of the gene blocks in which the 7-deoxyloganetin glucosyltransferase-like genes located were employed. The Ks for evaluating the relative divergence time of each family was acquired from PGDD (http://chibba.agtec. uga.edu/duplication/) [75]. Divergence time, T = Ks/2λ, with λ = 9.1*10^− 9^, which was derived from black cottonwood [76].

### Phylogenetic analysis

The phylogenetic trees in our analysis were constructed using RAxML. Sequence alignments were performed by multiple sequence comparison by log-expectation (Muscle) with default parameters. Trees made with codeml were constructed by coding sequences. Other trees were constructed by amino acid sequences. For trees constructed by amino acids, model PROTGAMMA was selected and for trees constructed by nucleotides, model ASC_BINGAMMA was selected. The presentation of trees was done by the Interactive Tree of Life (http://itol.embl.de) [77] and EvolView (http://www.evolgenius.info/evolview) [78].

### Expression divergence analysis

Six expression profiles of pollinated pistils of ‘Jinzhui x Yali’ and ‘Jinzhui x Jinzhui’ were used for expression divergence analysis. The divergences in the expression of three types of gene pairs were calculated separately: (1) When gene a and gene b were in one gene pair, their expression RPKM in six libraries was set as two matrices, A and B, where A = [a_k_] (k = 1~ 6) and B = [b_k_] (k = 1~ 6). (2) Expression conservation (EC) was calculated by EC (i) = PCC (A, B). (3) The expression divergence of gene pair ‘i’ was computed as 1-EC (i). All calculations were performed by R.

### RPKM values from RNA-seq of HRGPs in pollen and pollinated pistils

The RPKM values of *HRGPs* in pollen developmental stages and fruit developmental stage were acquired from our previous reports [79, 80]. The pollens of ‘Jinzhui’ and ‘Dangshansuli’ were collected from Jiangpu farm of Nanjing Agricultural University. To calculate the RPKM values of HRGPs in pollinated pistils of ‘Jinzhui x Yali’ and ‘Jinzhui x Jinzhui,’ the pistils of ‘Jinzhui’ and ‘Yali’ were all pollinated with ‘Jinzhui’ pollen and were collected in 24 h, 48 h and 72 h post-pollination in Jiangpu farm. All RPKM values were normalized into − 3 to 3 and displayed as heat maps by R. The expression pattern of HRGPs during the dynamic pear fruits development were acquired from NCBI and the RPKM values were normalized into − 4.55 to 4.55 and displayed as heat maps by R.

### qRT-PCR analysis

Total RNA used for qRT-PCR analysis was extracted from six different stages of pistil pollination (i.e. JY24, JY48, JY72, JJ24, JJ48 and JJ72). Total RNA was adjusted to the same concentration for first-strand cDNA synthesis using TransScript One-Step gDNA Removal and cDNA Synthesis SuperMix (TransGen Biotech Co. Ltd.) according to the manufacturer’s instructions. qRT-PCR analysis was carried out using LightCycler 480 SYBR GREEN I Master mix (Roche) according to the manufacturer’s protocol. We performed each reaction using a 20 μl mixture containing 12 μl of LightCycler 480 SYBR GREEN I Master, 5 μl of nuclease-free water, 1 μl of each primer and 1 μl of diluted cDNA. The qRT-PCR began with 5 min at 94 °C, followed by 55 cycles at 94 °C for 3 s, 60 °C for 10 s and 30s of extension at 72 °C. *Pyrus Tubulin* (accession no. AB239681) was used as the internal control gene and the relative expression levels were calculated with the 2^-ΔΔCt^ method. All primers were shown in Additional file 1: Table S4. RPKMs of *Pbr036330.1* and *Pbr010506.1* in pollinated pistils were standardized to their corresponding relative expressions in pollinated pistils.

### Identification of cis-regulated elements in HRGPs

To identify the potential transcription factor in the promoter sequences of HRGPs, TSSPlant in Softberry Website (http://www.softberry.com/) was used for this analysis [81]. We extracted the 1000 bp upstream sequences of HRGPs from WGD events and screened against the TSSPlant database.

## Additional files

Additional file 1: **Table S1.** Genomic annotations and classifications of *HRGPs* in pear. SP refers to signal peptide; GPI refers to glycosylphosphatidylinositol. **Table S2.** The transcription factor shared by HRGP gene pairs from WGD event. **Table S3.** The comparison of searching strategy between our method and the method previously reported. **Table S4.** Primers for *HRGPs* in qRT-PCR. (XLSX 114 kb)

Additional file 2: **Figure. S1.** Protein sequences of representative HRGPs in pear. Coloured sequences indicate the predicted signal peptides (pink) in the N-terminus, GPI anchors (light green) in the C-terminus, PA, AP, SP and TP repeats (yellow), lysine-rich regions (light blue), H1 tags (blue), SP_n_ motifs (dark blue) and PPV[X][KC] motifs (light pink). (PDF 4458 kb)

Additional file 3: **Figure. S2.** Phylogenetic tree of the EXT family. The phylogenetic tree was constructed by RAxML using amino acid sequences. Taxa with stars indicate SP3-EXT, with gcircles indicate SP4-EXT and with triangles indicate SP5-EXT. (PDF 2349 kb)

Additional file 4: **Figure. S3.** Phylogenetic tree of the PRP family. The phylogenetic tree was constructed by RAxML using amino acid sequences. The red taxa indicate PRPs with PPV[X]C motifs and the blue taxa indicate PRPs with PPV[X]K motifs. (PDF 2910 kb)

Additional file 5: **Figure. S4.** Heatmap of the expression levels of HRGP genes in reproductive tissues. A. The expression levels of HRGP genes in pollen. MP, HP, PT and SPT correspond to four different developmental stages: matured pollen, hydrated pollen, growing pollen tubes after three hours of hydration and stopped growing pollen tubes, respectively. B. The expression levels of HRGP genes in the pistils of ‘Jinzhui’ pollinated with self- and non-self-pollen; JY24, JJ24, JY48, JJ48, JY72 and JJ72 refer to pollinated pistils corresponding to time after pollination. The colour scale represents log2 transformed reads per kilobase per million (RPKM) values. Light green indicates low expression and red indicates high expression. (PDF 313 kb)

Additional file 6: **Figure. S5.** Heatmap of the expression levels of *HRGP* in 6 stages of pear fruit. S1~S6 correspond to the dynamic stages of fruit development at 15 d, 36 d,80 d, 110 d, 145 d and 167 d after flowering. (PDF 388 kb)

Additional file 7: **Figure. S6.** Relative expression levels of *HRGPs* in pollinated pistils. A-C. The relative expression levels of *Pbr036330.1* (A), *Pbr010506.1* (B) and *Pbr031110.1* (C) in different pear tissues. JY24, JJ24, JY48, JJ48, JY72 and JJ72 refer to pollinated pistils corresponding to time after pollination with ‘Yali’ and ‘Jinzhui’ pollen. (PDF 183 kb)

## Abbreviations

120 K: 120 kDa glycoprotein
A: Alanine
AGP: Arabinogalactan-protein
ECM: Extracellular matrix
EXT: Extensin
HMM: Hidden markov model
HRGP: Hydroxyproline-rich glycoprotein
Ka: Nonsynonymous substitutions per site
Ks: Synonymous substitutions per site
Muscle: Multiple sequence comparison by log-expectation
P: Proline
PCC: Pearson correlation coefficient
PELPIII: Class III pistil-specific extensin-like
RPKM: Reads per kilobase per million
SI: Self-incompatibility
T: Threonine
TTS: Transmitting tissue-specific
WGD: Whole genome duplication
