# Supplementary figures and images for: The unique evolutionary pattern of the Hydroxyproline-rich glycoproteins superfamily in Chinese white pear (Pyrus bretschneideri)
Source: BMC Plant Biol. 2018 Feb 17;18:36. doi: 10.1186/s12870-018-1252-2 (PMC5816549; doi:10.1186/s12870-018-1252-2)

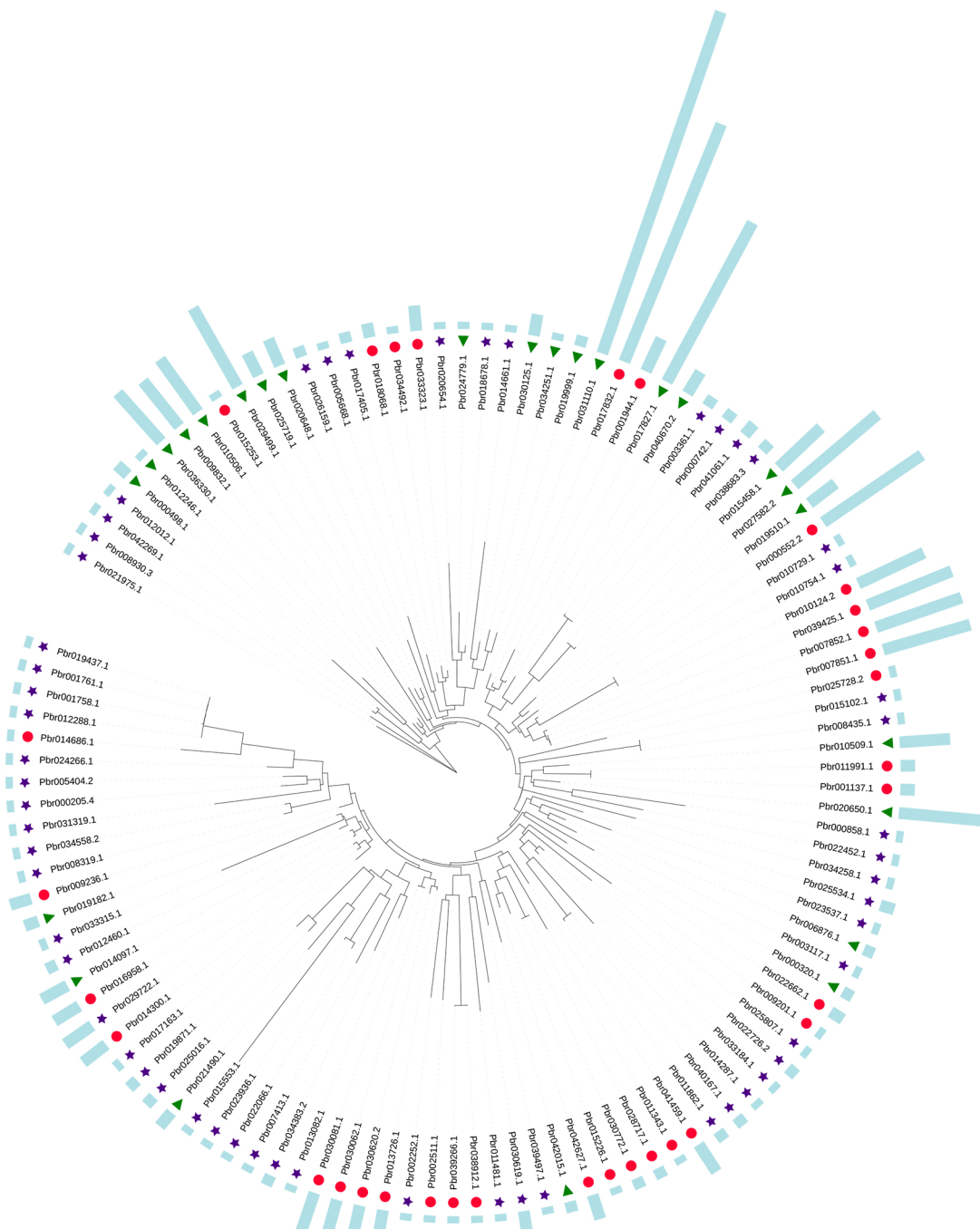

Supplement: Supplementary file 3 — Figure. S2. Phylogenetic tree of the EXT family. The phylogenetic tree was constructed by RAxML using amino acid sequences. Taxa with stars indicate SP3-EXT, with gcircles indicate SP4-EXT and with triangles indicate SP5-EXT. (PDF 2349 kb) [file 12870_2018_1252_MOESM3_ESM.pdf]

Tree scale: 1

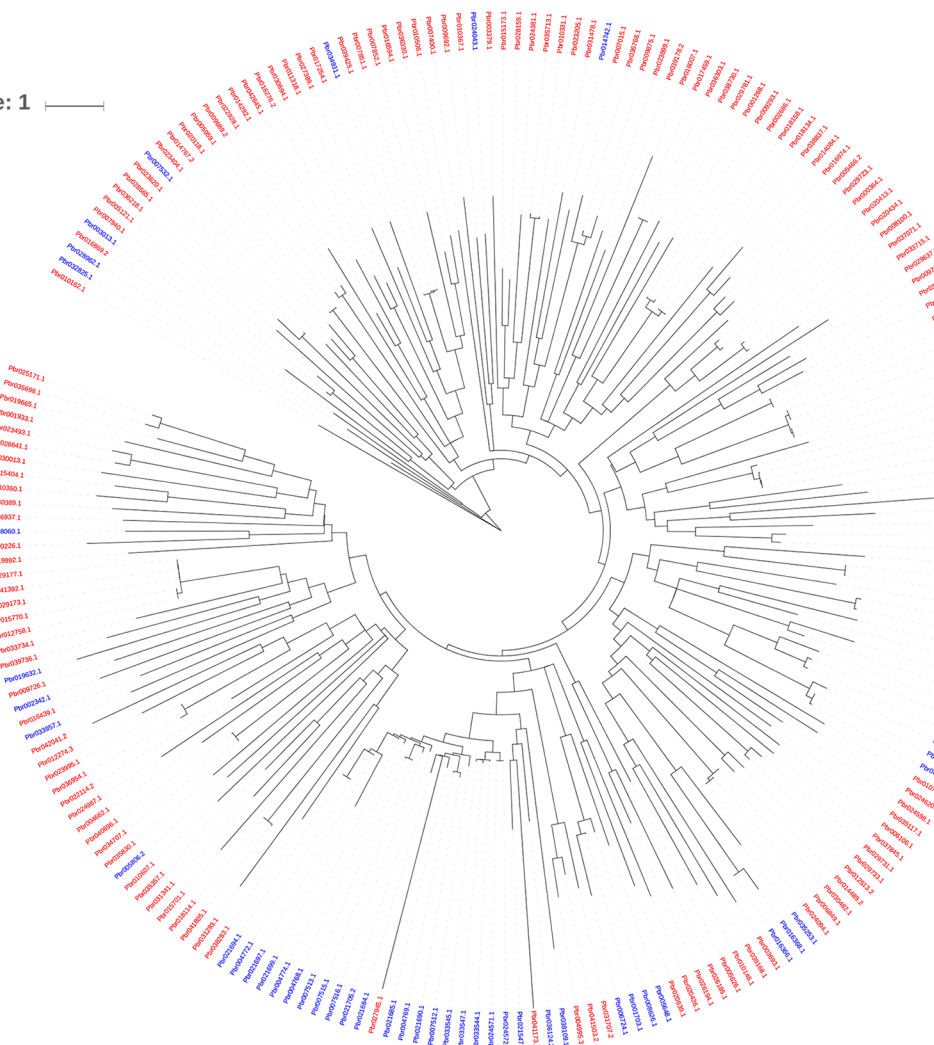

Supplement: Supplementary file 4 — Figure. S3. Phylogenetic tree of the PRP family. The phylogenetic tree was constructed by RAxML using amino acid sequences. The red taxa indicate PRPs with PPV[X]C motifs and the blue taxa indicate PRPs with PPV[X]K motifs. (PDF 2910 kb) [file 12870_2018_1252_MOESM4_ESM.pdf]

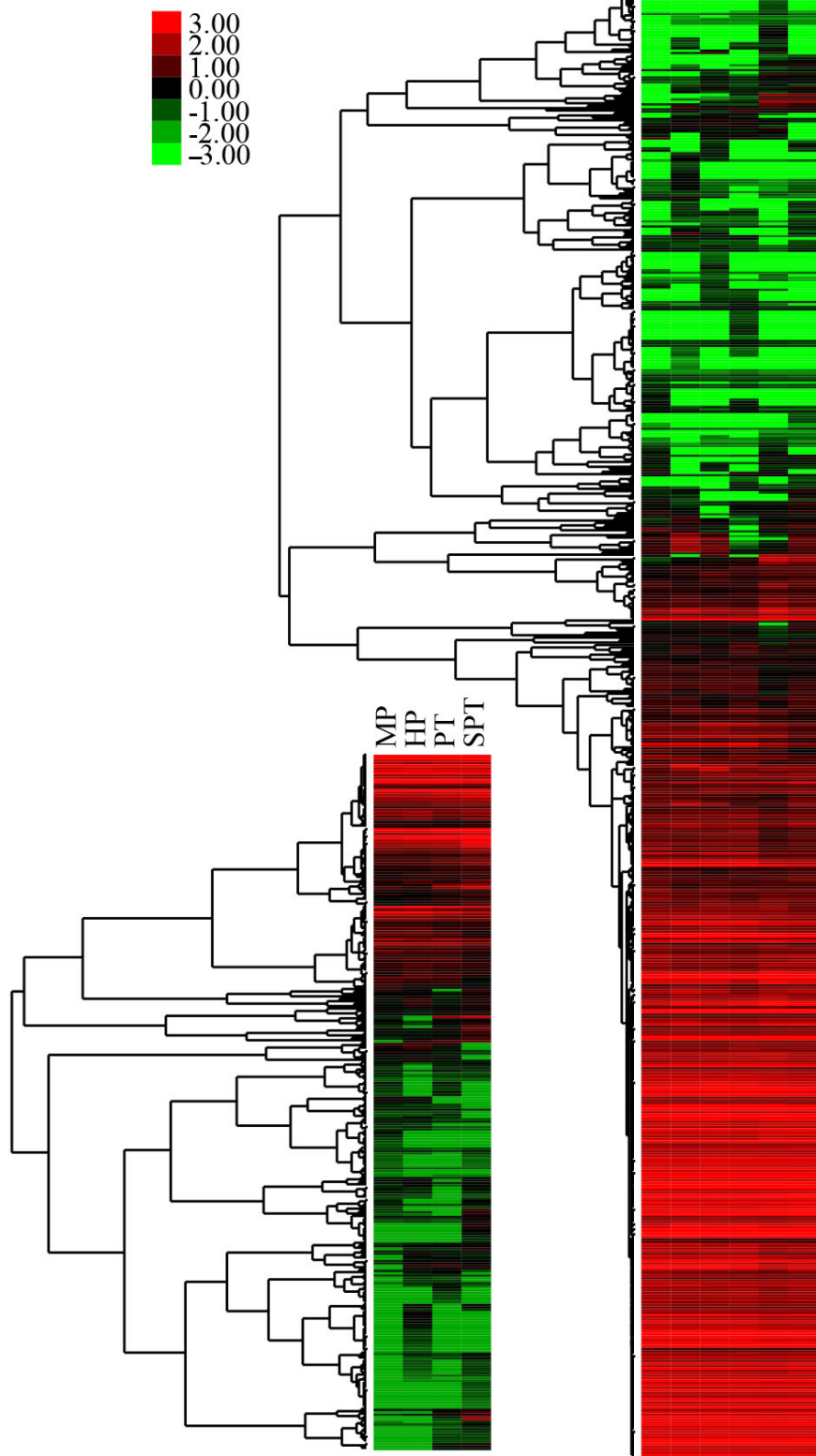

A

B

Supplement: Supplementary file 5 — Figure. S4. Heatmap of the expression levels of HRGP genes in reproductive tissues. A. The expression levels of HRGP genes in pollen. MP, HP, PT and SPT correspond to four different developmental stages: matured pollen, hydrated pollen, growing pollen tubes after three hours of hydration and stopped growing pollen tubes, respectively. B. The expression levels of HRGP genes in the pistils of ‘Jinzhui’ pollinated with self- and non-self-pollen; JY24, JJ24, JY48, JJ48, JY72 and JJ72 refer to pollinated pistils corresponding to time after pollination. The colour scale represents log2 transformed reads per kilobase per million (RPKM) values. Light green indicates low expression and red indicates high expression. (PDF 313 kb) [file 12870_2018_1252_MOESM5_ESM.pdf]

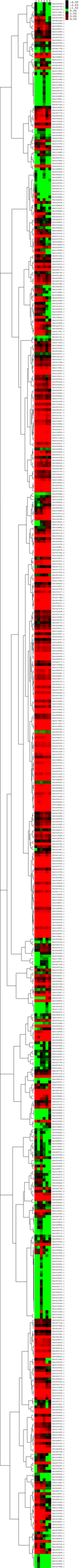

Supplement: Supplementary file 6 — Figure. S5. Heatmap of the expression levels of HRGP in 6 stages of pear fruit. S1~S6 correspond to the dynamic stages of fruit development at 15 d, 36 d,80 d, 110 d, 145 d and 167 d after flowering. (PDF 388 kb) [file 12870_2018_1252_MOESM6_ESM.pdf]

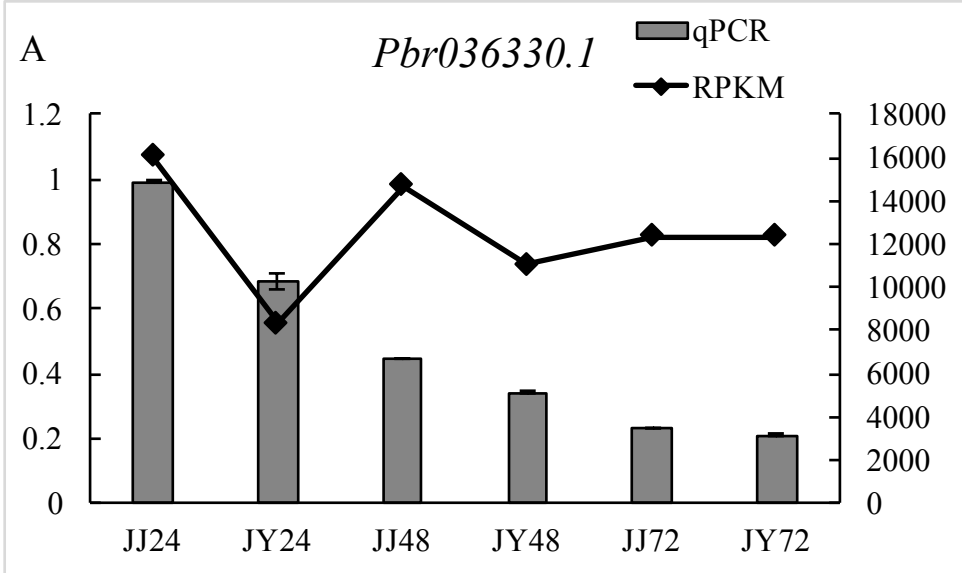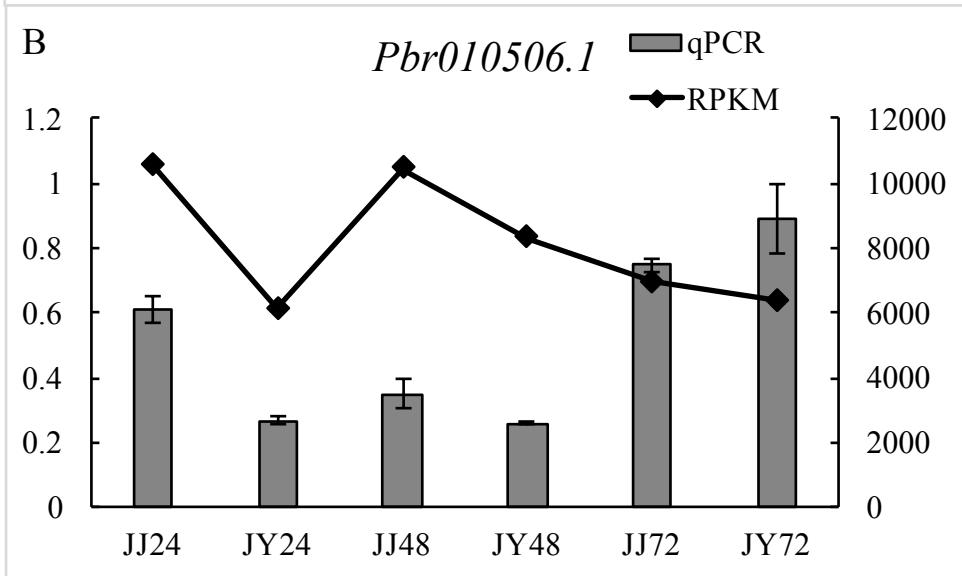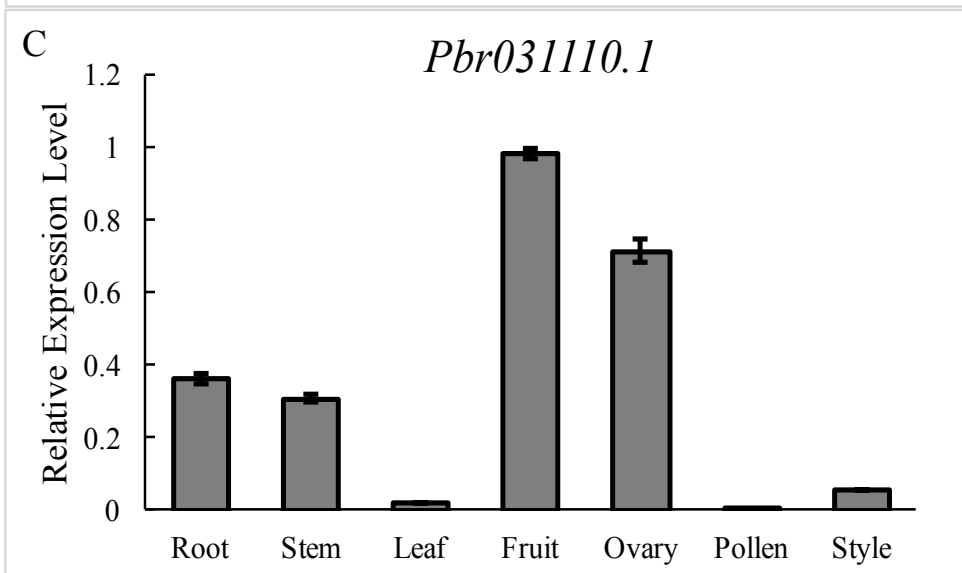

Supplement: Supplementary file 7 — Figure. S6. Relative expression levels of HRGPs in pollinated pistils. A-C. The relative expression levels of Pbr036330.1 (A), Pbr010506.1 (B) and Pbr031110.1 (C) in different pear tissues. JY24, JJ24, JY48, JJ48, JY72 and JJ72 refer to pollinated pistils corresponding to time after pollination with ‘Yali’ and ‘Jinzhui’ pollen. (PDF 183 kb) [file 12870_2018_1252_MOESM7_ESM.pdf]
