# Supplementary material for: The unique evolutionary pattern of the Hydroxyproline-rich glycoproteins superfamily in Chinese white pear (Pyrus bretschneideri)
Source: BMC Plant Biol. 2018 Feb 17;18:36. doi: 10.1186/s12870-018-1252-2 (PMC5816549; doi:10.1186/s12870-018-1252-2)
Supplement: Supplementary file 2 — Figure. S1. Protein sequences of representative HRGPs in pear. Coloured sequences indicate the predicted signal peptides (pink) in the N-terminus, GPI anchors (light green) in the C-terminus, PA, AP, SP and TP repeats (yellow), lysine-rich regions (light blue), H1 tags (blue), SPn motifs (dark blue) and PPV[X][KC] motifs (light pink). (PDF 4458 kb) [file 12870_2018_1252_MOESM2_ESM.pdf]

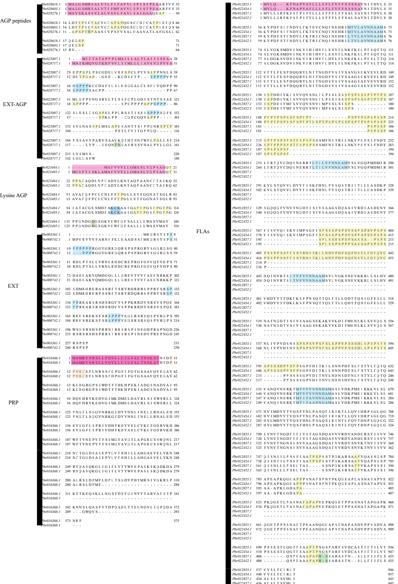
AGP peptides
EXT-AGP
Lysine AGP
EXT
PRP
FLAs
